# Do Reef Fish Habituate to Diver Presence? Evidence from Two Reef Sites with Contrasting Historical Levels of SCUBA Intensity in the Bay Islands, Honduras

**DOI:** 10.1371/journal.pone.0119645

**Published:** 2015-03-25

**Authors:** Benjamin M. Titus, Marymegan Daly, Dan A. Exton

**Affiliations:** 1 Department of Evolution, Ecology, and Organismal Biology, The Ohio State University, Columbus, Ohio, United States of America; 2 Operation Wallacea, Lincolnshire, England, United Kingdom; Dauphin Island Sea Lab, UNITED STATES

## Abstract

Contact between humans and the marine environment is increasing, but the capacity of communities to adapt to human presence remains largely unknown. The popularization of SCUBA diving has added a new dimension to human impacts in aquatic systems and, although individual-level impacts have been identified, cumulative effects on ecosystem function and community-wide responses are unclear. In principle, habituation may mitigate the consequences of human presence on the biology of an individual and allow the quick resumption of its ecological roles, but this has not been documented in aquatic systems. Here, we investigate the short-term impact of human presence and the long-term habituation potential of reef-fish communities to recreational SCUBA divers by studying symbiotic cleaning interactions on coral reefs with differing levels of historical contact with divers. We show that incidences of human contact result in a smaller decline in ecosystem function and more rapid resumption of baseline services on a reef in Utila, Honduras that has heavy historical levels of SCUBA diver presence, compared to an un-dived reef site in the Cayos Cochinos Marine Protected Area (CCMPA). Nonetheless, despite the generally smaller change in ecosystem function and decades of regular contact with divers, cleaning behavior is suppressed by >50% at Utila when divers are present. We hypothesize that community-wide habituation of reef fish is not fully achievable and may be biologically restricted to only partial habituation. Differential responses to human presence impacts the interpretation and execution of behavioral research where SCUBA is the predominant means of data collection, and provides an important rationale for future research investigating the interplay between human presence, ecosystem function, and community structure on coral reefs.

## Introduction

Human presence can have a major impact on animal behavior and through this have significant consequences for ecosystem health and function. Habituation and tolerance mitigate the consequences of this impact on the biology of individuals and their ecological roles by determining when, if, and how organisms resume natural behaviors [1, 2]. While the impacts of destructive human activity are more obvious, mere presence can lead to human-induced rapid environmental change, affecting biodiversity [3, 4], individual activity level [5], foraging behavior [6], nest success [1], flight response [7–9], hormone levels [10], anti-predator behavior [11, 12], habitat use [13], and aggression [14].

The majority of studies of animal behavior involve direct human observation. Minimally, this entails passive human presence, relying on the assumption that observers have a neutral impact on the behavior of interest. These qualifications can be exceedingly difficult to meet underwater. Often SCUBA divers appear suddenly and are suspended in the water column, looming over the benthos. SCUBA traditionally relies on noisy open-circuit regulators that emit low-frequency sound waves detectable up to 200m away [15, 16], and as visibility rarely exceeds 30m, divers must get close to the wildlife. This underwater “observer effect” has been acknowledged in scientific [17, 18] and recreational [19] contexts. For highly mobile organisms such as reef fish, studies of the impact of human presence have demonstrated an effect on flight distance, foraging patterns, diversity, and abundance [3, 4, 9, 10, 20–22], although these studies typically identify responses to one-off human contact events. We have yet to understand the long-term cumulative effect SCUBA diver presence has on animal behavior in the marine environment, especially the consequences of repeated exposure on ecosystem function, species interactions, and the habituation potential to diver presence across a community of individuals.

Services provided mutualistically are an integral part of the complex web of interactions in many communities and help maintain ecosystem health and function. On coral reefs, cleaning interactions are classic interspecific mutualisms between cleaners (i.e. gobies, wrasse, and shrimp) and a community of client fish that span the taxonomic spectrum [23–29]. The interactions between cleaners and client fish positively impact fish diversity and health [26, 30]. Cleaner species occupy discreet microhabitats that serve as cleaning stations that are intentionally sought by clients [26, 29]; at these cleaning stations, the client fish pose motionless and are vulnerable to predation while cleaners inspect and remove parasites from gills, mouth, and scales. Because cleaning is risky to the participants, involves multiple species that may differ in their sensitivity to diver presence, and is important for ecosystem function [23–29], these interactions are ideal for testing hypotheses about community-wide impacts of human presence.

On Caribbean coral reefs, the corkscrew sea anemone *Bartholomea annulata* and Pederson’s cleaner shrimp *Ancylomenes pedersoni* form ecologically important cleaning stations. These stations are small and stationary, easy to locate, and service members of at least 20 families of reef fish [23, 29, 31]. We use cleaning interactions at *B*. *annulata* stations as an indicator of community-level behavioral stability to investigate how SCUBA diver presence impacts the provision of this service in the focal ecosystems. We use (i) traditional visual diver observations (direct presence), (ii) remote videography with divers nearby (ambient presence), and (iii) remote videography alone (diver absence) to quantify reef fish cleaning frequency and duration of temporary cleaning disruption. We then explore whether prolonged historical exposure to diver presence leads to community-wide habituation by comparing two separate but similar reef sites in Honduras which have experienced different extremes of historical diving pressure.

We use this approach to demonstrate that complete habituation of reef fish to diver presence may be unattainable at the community level, regardless of the frequency and intensity of historical human contact and the opportunity for habituation it brings. Instead, we suggest that reef fish communities represent a mosaic of individuals that vary in their habituation levels and tolerance to divers. Our results bear on the interpretation of past ethological studies where direct human observation using SCUBA was the predominant form of data collection, and provide an important null hypothesis for future research on fish behavior and the impact of human presence on ecosystem function on coral reefs. The habituation potential of reef fish communities thus raises an important consideration that the ability to tolerate human presence and to maintain natural behaviors during episodes of diver contact could provide a competitive advantage to certain individuals and species, acting as a novel cue for natural selection and potentially sculpting reef fish communities in the future.

## Methods

### Study sites

We focus on two coral reef sites in the Bay Islands, Honduras. Coral View reef on the island of Utila (16°05’17.87”N 86°54’38.56”W) is a sloping fringing-reef extending from ca. 3–30m. It has been dived heavily and regularly from shore by recreational SCUBA divers for more than 20 years, and serves as the house reef for the adjacent Coral View Dive Resort. The site sees low level recreational boat traffic and snorkelers make regular visits alongside SCUBA divers. The second reef site is an unnamed reef in the Cayos Cochinos Marine Protected Area (CCMPA; 15°57’00.55”N 86°29’49.19”W) and is a sloping fringing-reef extending ca. 1.5–20m which, to the best of our knowledge, has never previously been dived. This reef is unbuoyed and unmarked in an area with heavy historical restrictions on diving geared towards clearly identified dives sites, and consultation with park managers at the Honduran Coral Reef Foundation (HCRF) confirmed no diving is known to have occurred there. Additionally, reef development within CCMPA is most extensive along the northern edge of the archipelago, where the majority of mooring buoys and dive sites are located. Thus, access to our study site is restricted by HCRF and is geographically separate from any recreational or scientific diving that occurs within CCMPA. Some infrequent boat traffic (small recreational) occurs close to the reef site, but no other historical contact with humans has taken place.

Both reef systems were surveyed for key ecosystem indicator criteria to assess their suitability for comparison in this study. These assessments all involved randomly placed 50m transects conducted at 6–8m depth in triplicate (n = 3) at six sub-sites within each system to provide local averages and to account for transiency in fish populations. Broad benthic community health was quantified through percentage live scleractinian coral cover, with data collected by point intercept using 0.25m intervals. Fish abundance and species richness were quantified using underwater visual census (UVC), with transect dimensions of 5 x 5m. Belt transects of 5m width were used to quantify density of *B*. *annulata*. All reef site assessments were conducted in tandem with cleaning observations, but were completed during the mid-morning or afternoon in order to avoid direct interference with diver observations and camera deployments at dawn. Assessments were completed in fewer than five dives at the study sites themselves, and so led to minimal human contact, especially when compared to the intense historical contact at Utila. Variations in key reef characteristics were statistically tested using independent t-tests in the software package SPSS [32].

### Observation of cleaning interactions

At each study site, all *B*. *annulata* between 4–9m depth hosting *A*. *pedersoni* were tagged, measured, and mapped to avoid duplication, and the number of shrimps recorded. Each station was then randomly assigned to one of two observational treatments: diver observed (Utila *n* = 31; CCMPA *n* = 31) or video observed (Utila *n* = 13; CCMPA *n* = 19). Diver observations lasted 20 min, after a 5 min acclimation period, from 3–5m distance [23, 27] using open-circuit SCUBA. Client fish were identified to family level and the number and length of each clean recorded following the protocol established by Huebner and Chadwick [29]. Video observations used GoPro Hero3 cameras mounted 1–2m from the anemone, recording continuously at 720p video quality to extend battery life (ca. 120–180 min). Both treatments occurred at dawn to coincide with the widely accepted period of maximum cleaning rates [29]. Although peak diving intensity at Utila occurs between 9am—3pm daily, dawn diving is also a regular occurrence and so our observation time is a common time for human contact. To ensure video cleaning data were analyzed in an equivalent and directly comparable manner to diver observations 20-minute blocks of video were analyzed using the same criteria as diver observations. Here, videos were analyzed 60 minutes into each recording to ensure that all divers had long exited the water. To test the null hypothesis that there is no significant difference in fish cleaning rates between diver collected and video collected data regardless of the historical diver presence all cleaning data from each station were compiled into counts (number of cleans per 20 min), and tested for a two-way interaction effect between reef site and treatment. Data were analyzed using a Poisson distributed Generalized Linear Model with a logarithmic link function, scalar value of 1, and Bonferroni correction for pairwise comparisons. The model was constructed using cleaning frequency as the dependent response variable, with two main-effects (site and treatment). Interaction effects were tested between reef site and treatment, with two treatment levels (direct diver observation and diver absence video observation) and two reef sites reflecting variation in historical diver presence (heavily dived: Utila and undived: Cayos Cochinos).

While the above analysis tested the effect of a SCUBA diver positioned directly in front of a cleaning station, ambient diver presence (defined here as a diver on a reef but > 10m from a cleaning station) may also have an effect on cleaning rates. In theory, divers may have a “halo-effect,” disrupting ecosystem function within an unknown radius, which may decrease in effect the further away a diver becomes. In our example, habituation to diver presence would reduce this halo-effect, allowing for cleaning rates to resume to ‘diver absence’ baseline rates even while divers remain in the water. To assess this halo-effect, videos at each station were separated into two treatments to provide separate measures of cleaning behavior in response to variation in diver presence: 1) ambient diver presence, and 2) diver absence. During ambient presence treatments, divers remained nearby on the reef (>10m from cleaning station); their open-circuit SCUBA was audible on recordings. Ambient presence video data were analyzed 5 minutes into each recording to provide an analogous “acclimation” time to mimic direct observation treatments. Diver absence videos were analyzed 60 minutes into each recording as above. Thus, data from each station represented repeated measures and significance between treatments was determined at each reef site independently using Wilcoxon signed-rank tests (non-parametric paired t-test).

To provide a third measure of ecosystem function disruption in response to diver presence, we recorded the interval of time (minutes) from camera deployment until the first cleaning interaction. As above, the comparison of these intervals was conducted in a repeated measures design using two treatments at each reef site: 1) ambient diver presence and 2) diver absence. Divers remained on the reef (> 10m from cleaning station) during ambient presence treatments; cameras were re-deployed at the same station by free-divers who immediately exited the water for diver absence treatments. This design tested the hypothesis that habituation shortens the avoidance response to human presence and permits a more rapid return to natural ecosystem functioning. This component used footage from both reef sites (Utila *n* = 11; CCMPA *n* = 15). Only cleaning stations where cleaning interactions were previously recorded could be used for this analysis, thus reducing our sample size. Statistical significance was assessed using non-parametric Wilcoxon signed-rank tests (non-parametric paired t-test) as above.

While we acknowledge that cleaning interactions are multi-partner mutualisms predicated on the cooperation and decision making of both partners, we interpret our data as though the effects of diver presence is entirely placed on fish clients and not on cleaner shrimp. Cleaner shrimp at both reef sites readily signaled their availability to clean to passing SCUBA divers and when approached crawled onto, and inspected, outstretched hands. Although some fish visits do not result in a “clean,” this willingness to inspect human hands signals that the decision by shrimp to clean a client fish is independent of diver presence or absence.

Finally, although replication at the “cleaner-station” level has provided us with results that we feel accurately reflect the long-term effects of human presence on reef fish communities at each reef site, only single replicate sites were used at the level of “historical human presence.” Given the difficulty in finding accessible reefs of such contrasting levels of human contact that are also comparable in terms of key ecosystem indicator criteria, this pseudoreplicated sampling design was unavoidable. While this may limit our ability to broadly generalize our results to other reef sites or ecosystems, we provide important hypotheses for future studies at other reef sites to test the generality of the data presented here.

All fieldwork was observational, non-extractive data collection, conducted under permit number 19985 issued by the Honduran government.

## Results

### Comparability of focal sites

**Fig 1:**
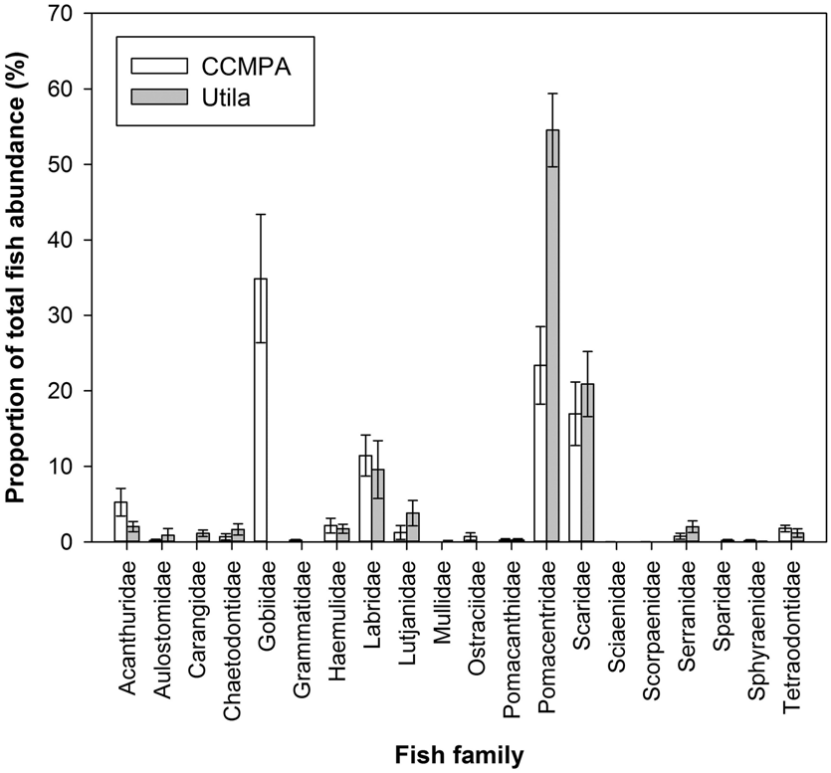
Coral reef fish community composition at study sites in the Cayos Cochinos Marine Protected area (CCMPA, white bars) and Utila (gray bars). Data presented represent the total proportion of fish abundance (means ± 1 SE) at the family level.

Despite major differences in historical diver presence, we find no significant differences between the two reef systems with respect to scleractinian coral cover (Utila = 19.54 ±2.45%; CCMPA = 21.06 ±1.44%), fish species richness (Utila = 31.06 ±1.44 species; CCMPA = 29.67 ±1.11 species), or *B*. *annulata* cleaning station density (Utila = 3.78 ±0.84 250m^-2^; CCMPA = 4.06 ±0.68 250m^-2^). Species from 18 fish families were documented at these sites (Utila: *n* = 15; CCMPA: *n* = 15) representing the potential reef fish client pool (Fig. 1). Total fish abundance was higher at CCMPA (Utila = 539.56 ±58.78 individuals 250m^-2^; CCMPA = 892.78 ±142.91 individuals 250m^-2^) (t(34) = 51.35, p < 0.01). There was no significant difference in anemone size (CCMPA: 141.48 ± 25.68 cm^2^; Utila: 136.98 ± 23.91 cm^2^; Mann-Whitney U-Test; U = 174.5, p = 0.85) or group size of *A*. *pedersoni* (CCMPA: 3.10 ± 0.42 shrimp; Utila: 2.80 ± 0.56 shrimp; U = 718.5, p = 0.22) between stations used across treatments. Importantly, total cleaning rates recorded via video observations (diver absence) were not significantly different between sites (CCMPA = 1.73 ± 1.82 cleans 20min^-1^; Utila = 2.61 ± 3.45 cleans 20min^-1^ U = 138, p = 0.58), indicating a similar natural baseline cleaning frequency, and validating our later comparisons of cleaning behavior in response to diver presence.

### Impact of diver presence on cleaning interactions

**Fig 2:**
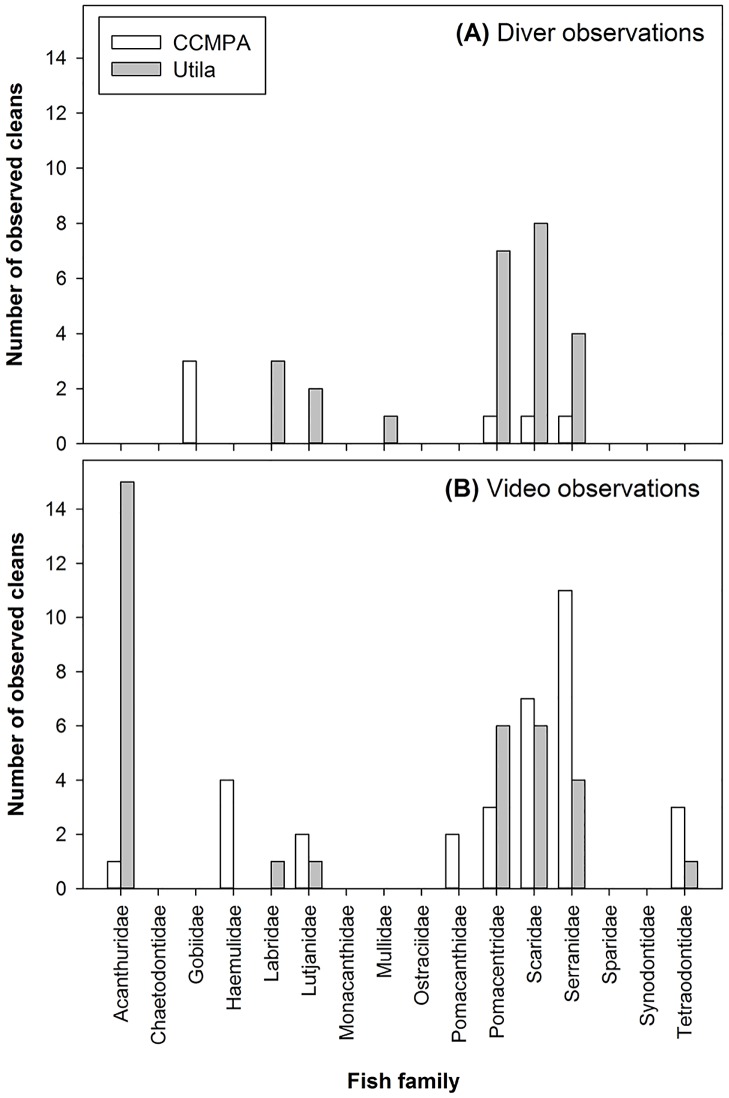
Variation in fish client diversity (family) observed during cleaning interactions. A) direct diver observatons and B) video observations at Cayos Cochinos Marine Protected area (CCMPA, white bars) and Utila (gray bars).

The presence of humans in the form of recreational SCUBA divers clearly altered the frequency of cleaning interactions taking place at both sites. At the previously un-dived CCMPA site, cleaning interactions were seldom observed directly by divers, resulting in a mean cleaning rate of only 0.19 ±0.11 20min^-1^. Divers at CCMPA only recorded six cleaning interactions from four client families at 31 directly observed cleaning stations (Fig. 2a). In contrast, video observations at CCMPA recorded 33 cleaning interactions from eight client families (Fig. 2b), resulting in a mean cleaning rate of 1.74 ±0.42 20min^-1^. At Utila, divers observed 25 cleaning interactions from six client families (Fig. 2a) resulting in a mean cleaning rate of 0.81 ±0.23 20min^-1^; four times greater than observed at CCMPA. Video observations at Utila recorded 34 cleaning interactions from seven client families (Fig. 2b) with a resulting cleaning rate of 2.62 ±0.96 20min^-1^. In general, the diversity of clients recorded at cleaning stations at both sites broadly reflect their respective abundances. Only Serranidae (groupers) and Acanthuridae (surgeonfish) appear over represented at our cleaning stations relative to their underlying abundance. Only Gobidae (gobies) appear underrepresented relative to its underlying abundance (Fig. 1 and 2).

**Fig 3:**
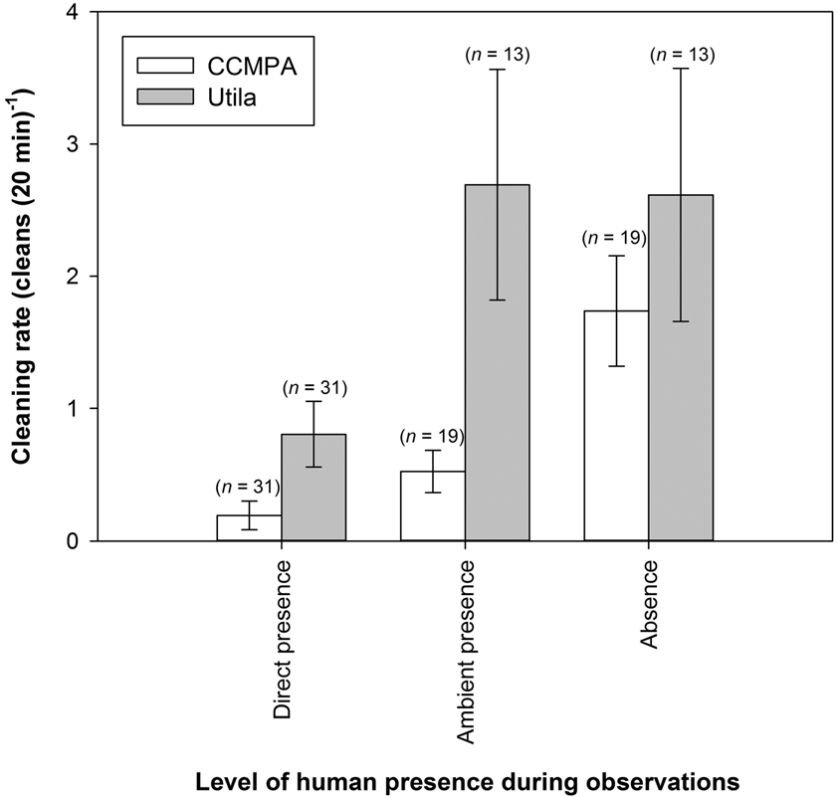
Rates of cleaning interactions (number of cleans 20min^-1^) between *Ancylomenes pedersoni* and their client fish across a gradient of diver presence treatments at Cayos Cochinos Marine Protected Area (CCMPA, un-dived reef) and Utila (heavily dived reef). Data show mean values ± standard error, where *n* refers to the number of independent anemone cleaning stations observed for each treatment (*n* ≥ 13). Cleaning rate is defined as the frequency of successfully completed cleaning interactions between shrimps and client fish species over a given time period.

Direct comparisons between diver (direct presence) and video (diver absence) treatments revealed a significant overall test result (Poisson distributed Generalized Linear Model: d.f. = 3, c^2^ = 44.83, p < 0.001), and a significant interaction effect between reef site and treatment (Site*Treatment: c^2^ = 3.889, p < 0.05). Bonferroni-corrected pairwise comparisons revealed a gradient of significant diver impacts across all treatments (Fig. 3). For both sites, the rates of cleaning interactions in the absence of divers were significantly higher than those observed directly by divers (Utila diver vs Utila video, p < 0.005; CCMPA diver vs CCMPA video, p < 0.001; Fig. 3). Additionally, at the heavily dived Utila reef, diver observations yielded a cleaning rate four times higher than diver observations at CCMPA (p < 0.005), yet was significantly less than video cleaning rate at CCMPA (p < 0.05; Fig. 3). Although direct diver presence showed a clear signature of suppressing cleaning interactions at both sites, the effect of ambient diver presence on reef fish cleaning rates differed between Utila and CCMPA. At CCMPA, cleaning rate with ambient presence was similar to that of direct diver observations (0.53 ±0.16 20min^-1^), and significantly less than diver absence (Wilcoxon signed-rank test; W = 66, p < 0.05; Fig. 3). In contrast, at Utila the frequency of cleans during ambient diver presence was not significantly different than during total diver absence (2.70 ±0.87 20min^-1^; Fig. 3), indicating a potential reduction in a diver’s “halo-effect” on reef fish behavior at Utila but not at CCMPA.

**Fig 4:**
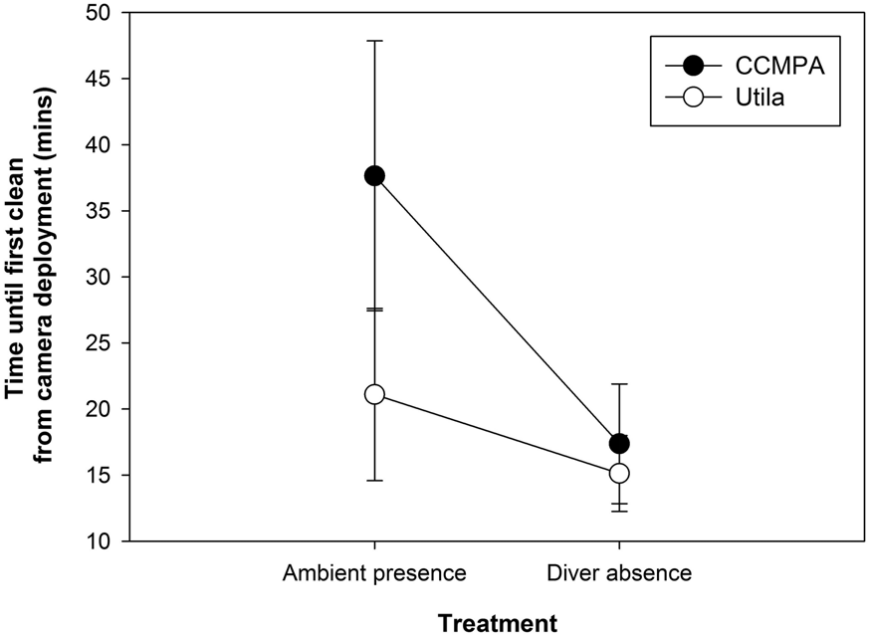
Mean length of time (min) from camera deployment to first successfully completed cleaning interaction at Cayos Cochinos Marine Protected Area (CCMPA, un-dived reef), and Utila (heavily dived reef) with passive ambient SCUBA diver presence and SCUBA diver absence. Ambient diver presence is here defined as SCUBA divers in the water during video observations, at least 10m distant from the observed anemone station, with bubbles are audible on video footage.

Similarly, cleaning behavior resumed significantly faster at CCMPA with diver absence (8.43 ±1.57 min) than with ambient diver presence (43.47 ±10.96 min; W = 108, p < 0.005; Fig. 4). At Utila, there was no significant difference in the amount of time before cleaning behavior resumed regardless of ambient diver presence (21.06 ±6.51 min) or diver absence (15.13 ±2.87 min; W = 25, p = 0.21; Fig. 4). Across all trials, the observed suppression of cleaning rates by divers increased as the intensity of data collection treatment increased (direct diver à ambient diver à total absence), and as historical levels of diver presence decreased (Fig. 3 and 4).

All data collected and analyzed in the completion of this manuscript have been provided as a Supplementary Table (S1 Table).

## Discussion

Taken together, our findings suggest that SCUBA divers have a clearly defined impact from SCUBA diver presence on natural ecosystem function in the Bay Islands, Honduras. This impact is less pronounced at locations that have higher intensity and regularity of historical levels of diver presence. Virtually no cleaning interactions were observed directly by divers at CCMPA, despite statistically similar cleaning station abundance to Utila, and a similar client pool. These results indicate a consistent avoidance response to SCUBA divers across the entire community of reef fish, regardless of taxonomy or trophic position, variables that have been shown to affect responses of fish to SCUBA divers in other systems [8, 9, 33]. Divers observed significantly more cleaning on the heavily dived reef at Utila, although these diver-observed rates were still significantly depressed compared to those observed from the video data set. Thus, despite the generally positive relationship between historical levels of diver activity and resilience to diver presence, full habituation across all individuals and species of reef fish has not been achieved at Utila: direct diver presence continues to depresses cleaning rates.

Ambient diver presence treatments further refine our understanding of ecosystem function disruption as the mere presence of SCUBA divers on the reef at CCMPA depressed cleaning interactions and even retarded the resumption of cleaning activity. No such disruptions were observed at Utila: cleaning rates under ambient diver presence are indistinguishable from those in the absence of divers, and cleaning resumes quickly after diver disturbance, even if divers are nearby. Consequently, the repeated heavy exposure to SCUBA divers may have led to a reduction in the diver “halo-effect” on reef fish behaviors at Utila, but not at the undived CCMPA reef site. These data suggest that some level of habituation has occurred at Utila, yet direct diver observation data suggest that the reef fish community is only partially habituated to diver presence. Thus, community-wide habituation of reef fish to SCUBA diver presence is either an ongoing process spanning over 20 years of daily diver interaction, or an unachievable state. We argue the latter appears more likely.

The Utila reef has been dived regularly and heavily for more than two decades. As twenty years exceeds the normal lifespan of a reef fish, human presence has been a common feature of the ecosystem for at least one full generation. The communities in which these fish live are dynamic, with continual loss via mortality and emigration of individuals likely to be habituated and continual gain of un-habituated individuals via recruitment and immigration. If in close enough proximity to a heavily dived reef site, an undived reef may also acquire habituated individuals through immigration and, in theory, exhibit some level of tolerance to divers at the community level. Thus, reef fish communities likely represent a mosaic of individuals expressing varying levels of habituation. This explains the clear but diminished impact of diver presence on cleaning rate found on Utila compared to CCMPA and also the similar interval for resumption of cleaning between ambient presence and diver absence treatments. It also explains the higher variance in cleaning rates observed at Utila. This mosaic may therefore represent the full extent to which a reef fish community can habituate to SCUBA diver presence within generational timescales.

The nuances of reef fish habituation are unclear and, to date, we lack sufficient data that generate expectations as to how this occurs. Habituation could occur at the individual level, species level, or (likely) both. Some species may be inherently more tolerant of SCUBA divers, while individual learned experience in a naturally intolerant species could trump this intolerance and lead to habituation. Alternatively, or in conjunction, habituation may be similar to flight initiation distance (FID), which appears to be a function of body size, taxonomic ID, group size, and trophic level [8, 9, 33, 34]. Individual habituation or tolerance could then vary over time, becoming more or less tolerant at different life stages. Finally, fish habituation to diver presence could be a random process with respect to taxonomy and life stage, varying at the level of the individual but not in a consistent way at the level of e.g., species or life history stage.

Nested within the concept of individual “tolerance” is also individual variation in risk-taking behavior. Trade offs between activity level and resource use, especially in the face of predation pressure, can shape community dynamics [35, 36]. Here, parasite load and the perceived risk of SCUBA divers could contribute to individual variation in cleaning rates when divers are directly in front of cleaning stations. Detrimental parasite loading could conceivably outweigh the risk of predation and increase cleaning rates; fish with fewer parasites could afford to forgo cleaning and wait until it becomes less risky. Although we see no evidence of full habituation of the reef fish communities we study, we see five avenues through which full community-wide habituation could result: 1) ecosystems exhibit lower population turnover, or 2) human presence in the ecosystem is not limited by time constraints (i.e. SCUBA) and can therefore be considered “full time” (the most heavily dived reefs could realistically fit into this category). 3) Individuals within the ecosystem habituate rapidly to human presence through direct experience, or 4) individuals within the ecosystem respond to cultural cues (non-avoidance of humans is learned from others in the community). Finally, 5) individuals within the ecosystem do not change their tolerance, but sort into or out of an ecosystem based on tolerance to humans, with those unaffected by human presence remaining in the ecosystem and those affected immigrating to human-free habitats.

A logical extension of the conclusion that reef fish communities do not fully habituate to diver presence would suggest that the direct presence of SCUBA divers is sufficiently threatening to tip the balance against the benefit of parasite removal: fish forgo the procurement of cleaning services and accept the cost of reduced fitness as a result. Cleaning symbioses are well known to improve the fitness of client fish [26, 30], and a tradeoff can be assumed between the ecological benefits of ecto-parasite removal and the basic need to avoid predation. This tradeoff raises worrying questions about the long-term impact of ecotourism on coral reef ecosystem function, although our finding that cleaning resumes relatively quickly even on un-habituated reefs suggests this is at least partly negated by short-term adaptive processes amongst reef fish, reducing the overall cost to community fitness. It is unclear whether short-term acclimation to SCUBA diver presence provides an inherent selective advantage and thus drives habituation over evolutionary timescales, as SCUBA in offshore waters and at depth is a relatively new impact. However, a decrease in the acquisition of cleaning services with clearly defined benefits to health would suggest that a negative impact on the fitness and potentially lifespan of individuals could be expected.

Lastly, conclusions drawn from animal behavior studies involving direct human observations depend on the assumption that observers have a neutral impact on the behavior of interest. These observations minimally entail passive human presence, and based on our findings we encourage a reduction in reliance on traditional observational approaches to studying animals using SCUBA and the incorporation of sites with historically high levels of diver presence wherever possible. Considering our data, direct diver observations alone would have significantly underestimated the natural cleaning rate at both sites and led to a falsely assumed, albeit statistically supported, inference of a difference in cleaning rate between CCMPA and Utila that video observations show to be incorrect. This would have led to false conclusions about ecosystem function and to a misconception of the factors controlling and structuring these interactions.

Remote videography may not be suitable or practically applied to every question and diver presence may not impact all aspects of behavior. Regardless, the potential impact of divers should be incorporated more thoroughly within the context of experimental design, and our hypothesis that reef fish communities cannot fully habituate to SCUBA diver presence will provide an important null hypothesis for future research. Our results suggest that novel SCUBA diver presence on coral reef ecosystems will amplify disturbances associated with pressures such as coastal development and will retard the recovery of baseline ecosystem function, although repeated contact will ultimately lead to a reduction in the extent of this negative impact through partial habituation. However, the balance between the negative impacts of diver presence and the wide-ranging direct economic and conservation benefits of ecotourism remains to be seen, and a long-term net gain to the ecosystem is ultimately feasible. Regardless, our findings alter the way we think about interactions between humans and the marine environment, and suggest that unseen mechanisms are at play at the community level when coral reef ecosystems come into contact with SCUBA divers.

## Supporting Information

S1 Table Raw data collected and used for the analysis of this manuscript. Data included are from SCUBA diver collected and remote video collected cleaning interactions at Cayos Cochinos Marine Protected Area (CCMPA) and Utila.
(PDF)
