# Supplementary material for: Do Reef Fish Habituate to Diver Presence? Evidence from Two Reef Sites with Contrasting Historical Levels of SCUBA Intensity in the Bay Islands, Honduras
Source: PLoS One. 2015 Mar 25;10(3):e0119645. doi: 10.1371/journal.pone.0119645 (PMC4373863; doi:10.1371/journal.pone.0119645)
Supplement: S1 Table — Data included are from SCUBA diver collected and remote video collected cleaning interactions at Cayos Cochinos Marine Protected Area (CCMPA) and Utila. (PDF) [file pone.0119645.s001.pdf]

Cleaning Rate Diver vs Video

Reef Site	ID	Diver/Video/Ambient	Cleans/20min
CCMPA	93	Diver	1
CCMPA	90	Diver	0
CCMPA	91	Diver	0
CCMPA	92	Diver	0
CCMPA	73	Diver	0
CCMPA	72	Diver	0
CCMPA	77	Diver	0
CCMPA	97	Diver	0
CCMPA	13	Diver	0
CCMPA	96	Diver	1
CCMPA	74	Diver	0
CCMPA	78	Diver	0
CCMPA	64	Diver	0
CCMPA	6	Diver	0
CCMPA	65	Diver	0
CCMPA	5	Diver	1
CCMPA	77	Diver	0
CCMPA	16	Diver	0
CCMPA	82	Diver	0
CCMPA	70	Diver	0
CCMPA	7	Diver	0
CCMPA	62	Diver	0
CCMPA	66	Diver	0
CCMPA	57	Diver	0
CCMPA	81	Diver	0
CCMPA	56	Diver	0
CCMPA	17	Diver	0
CCMPA	45	Diver	0
CCMPA	55	Diver	0
CCMPA	51	Diver	0
CCMPA	4	Diver	3
CCMPA	83	Video	4
CCMPA	84	Video	0
CCMPA	100	Video	5
CCMPA	67	Video	0
CCMPA	61	Video	3
CCMPA	79	Video	0
CCMPA	1	Video	4
CCMPA	68	Video	4
CCMPA	3	Video	0
CCMPA	15	Video	2
CCMPA	18	Video	0
CCMPA	9	Video	0
CCMPA	20	Video	0
CCMPA	69	Video	1
CCMPA	14	Video	2

CCMPA	45	Video	3
CCMPA	2	Video	1
CCMPA	60	Video	0
CCMPA	80	Video	4
Utila	31	Diver	0
Utila	21	Diver	0
Utila	52	Diver	0
Utila	33	Diver	0
Utila	29	Diver	0
Utila	53	Diver	1
Utila	54	Diver	0
Utila	1	Diver	1
Utila	2	Diver	1
Utila	101	Diver	2
Utila	104	Diver	4
Utila	23	Diver	0
Utila	59	Diver	0
Utila	105	Diver	1
Utila	38	Diver	4
Utila	22	Diver	0
Utila	26	Diver	1
Utila	4	Diver	0
Utila	41	Diver	0
Utila	43	Diver	0
Utila	27	Diver	0
Utila	44	Diver	1
Utila	36	Diver	5
Utila	11	Diver	3
Utila	32	Diver	0
Utila	112	Diver	0
Utila	109	Diver	0
Utila	113	Diver	0
Utila	34	Diver	0
Utila	110	Diver	0
Utila	111	Diver	1
Utila	12	Video	0
Utila	25	Video	1
Utila	30	Video	0
Utila	32	Video	1
Utila	3	Video	4
Utila	35	Video	3
Utila	106	Video	0
Utila	108	Video	1
Utila	5	Video	4
Utila	107	Video	1
Utila	28	Video	3
Utila	102	Video	3
Utila	103	Video	13

Cleaning Rate Ambient Diver Presence vs Video

	ID	Ambient Presence	Diver Absence (video)
CCMPA	83	1	4
CCMPA	84	0	0
CCMPA	100	0	5
CCMPA	67	0	0
CCMPA	61	0	3
CCMPA	79	1	0
CCMPA	1	0	4
CCMPA	68	0	4
CCMPA	3	0	0
CCMPA	15	1	2
CCMPA	18	0	0
CCMPA	9	0	0
CCMPA	20	1	0
CCMPA	69	0	1
CCMPA	14	1	2
CCMPA	45	0	3
CCMPA	2	1	1
CCMPA	60	2	0
CCMPA	80	2	4

	ID	Ambient Presence	Diver Absence (video)
Utila	12	1	0
Utila	25	1	1
Utila	30	0	0
Utila	32	3	1
Utila	3	4	4
Utila	35	3	3
Utila	106	0	0
Utila	108	0	1
Utila	5	8	4
Utila	107	0	1
Utila	28	7	3
Utila	102	0	3
Utila	103	8	13

Time until first cleaning interaction from camera deployment

	ID	Divers Present	Divers absent
CCMPA	83	23.00	3
CCMPA	100	41.00	4.5
CCMPA	61	49.50	8
CCMPA	79	3.50	9
CCMPA	1	62.00	20
CCMPA	68	28.00	4.66
CCMPA	3	135.00	7

CCMPA	15	1.00	4
CCMPA	18	75.00	3
CCMPA	9	135.00	15.6
CCMPA	20	18.33	4.33
CCMPA	69	22.00	2.5
CCMPA	14	10.25	12
CCMPA	45	32.00	21
CCMPA	2	16.50	8

	**ID**	**Divers Present**	**Divers absent**
Utila	12	25.33	2
Utila	25	19.75	12
Utila	32	15	1
Utila	3	6.75	13
Utila	35	9	16
Utila	108	45	42
Utila	5	1	8
Utila	107	71	8
Utila	28	2	13
Utila	102	33	19
Utila	103	4	4
